# The Heart-Brain Team Approach in Patent Foramen Ovale Closure

**DOI:** 10.3389/fneur.2020.561938

**Published:** 2020-10-15

**Authors:** Fareed Moses S. Collado, Clifford J. Kavinsky

**Affiliations:** Rush University Medical Center, Chicago, IL, United States

**Keywords:** heart team approach, right to left cardiac shunt, stroke, interventional cardiac procedure, heart brain team, PFO closure device, embolic stroke, patent forame ovale

Modern medicine mandates a multi-disciplinary approach in treating complex diseases. In cardiology, the heart team approach is often applied to the treatment of patients with complex cardiac diseases.

Cardiologists have long been collaborating with other specialists. Oncologists and cardiologists have already merged into a novel sub-specialty called cardio-oncology in treating patients with heart disease and cancer. Vascular surgeons and interventional radiologists have historically competed with cardiologists in treating peripheral artery disease. However, in the current era, a more collaborative environment is becoming more evident. Subspecialty training in medicine has diverged the entire medical field into different modalities with each specialist tackling a very specific disease process. However, these diseases are oftentimes too complex to be managed by a single specialist. To date, stroke in the setting of a patent foramen ovale (PFO) is one of the only few disease processes wherein stroke neurologists and cardiologists closely collaborate.

The history of managing PFO for stroke prevention endured a long and arduous journey. Contradictory opinions by cardiologists and neurologists in managing patients with PFO created an oppositional relationship between the two specialties. This schism was fueled by the conflicting results of multiple randomized clinical trials for percutaneous PFO closure.

The CLOSURE I trial in 2012 and the PC trial in 2013 demonstrated similar, albeit disappointing results. The results showed a non-statistically significant trend toward benefit with closure device for secondary prevention of stroke compared with current medical therapy. These two trials on PFO closure created a profound impact in the United States. Since then, PFO closure was largely forgotten and was not supported by stake holder societies and third party payers (1).

The conflicting results of these trials also created such an impact in the field of neurology that in 2016, the guidelines of the American Academy of Neurology (AAN) discouraged the use of PFO closure for cryptogenic stroke (2).

The relationship gap between stroke neurologists and interventional cardiologists widened after the inconsistent results of the CLOSURE I and PC trial. It also commonly led to frequent debates and difference in opinions between both specialists. On the other hand, patients with PFO and cryptogenic stroke continued to be treated with anticoagulation or antiplatelet therapy without any effective alternative.

After the AAN recommendation, the results of the landmark trials from the RESPECT (long term follow up) and REDUCE trials were released in 2017. Both trials resurrected the use of PFO closure for stroke prevention. Both trials demonstrated superiority of PFO closure device over medical therapy in secondary stroke prevention. The results of both the RESPECT and REDUCE trials ultimately led to the FDA approval of the Amplatzer PFO Occluder (Abbott Structural, Santa Clara, CA) and the Gore Cardioform Septal Occluder (W. L. Gore and Associates, Inc., Newark, DE), respectively.

Since the 2016 AAN guidelines, there was an overwhelming consensus of the landmark PFO trials on the superiority of PFO closure over medical therapy alone in preventing recurrent ischemic stroke. Four years later, the AAN released a revised advisory regarding PFO closure. It states “*In patients younger than 60 years with a PFO and embolic-appearing infarct and no other mechanism of stroke identified, clinicians may recommend closure following a discussion of potential benefits (absolute recurrent stroke risk reduction of 3.4% at 5 years) and risks (periprocedural complication rate of 3.9% and increased absolute rate of non-periprocedural atrial fibrillation of 0.33% per year)”* (3). The release of this statement is both meaningful and historic. It not only acknowledged the results of the randomized clinical trials but also highlights the continued partnership between neurologists and cardiologists.

After the FDA approval of the two PFO closure devices, as well as the “blessing” of the AAN, it is anticipated that there will be a significant rise in PFO closure procedures in the next several years. However, cardiologists must be vigilant more than ever. Given that PFOs are present in approximately a third of the population, the risks of unnecessary procedures in patients who do not meet the indication for PFO closure should be strongly mitigated. Ensuring the appropriateness and delivery of patient-centered and quality care of our patients is critical. This goal, in our opinion, can only be achieved by the heart-brain team approach.

Patient selection is the single most important variable in effective and safe delivery of PFO treatment. Partnership with neurologists, specifically stroke neurologists, is a critical preliminary step in patient selection. A formal neurological consultation is mandatory prior to any PFO closure. In fact, no patient with PFO and stroke should undergo PFO closure without being evaluated by a stroke neurologist. A thorough evaluation of the possible etiology of stroke should be initiated. A battery of tests should be initiated by either the stroke neurologist or cardiologist including a transthoracic echocardiogram with a bubble study to rule out a right-to-left shunt, a heart rhythm monitor at least for 30 days to rule out atrial fibrillation, hypercoagulable work up, bilateral carotid ultrasound, and Doppler ultrasound to rule out lower extremity venous thrombosis.

Interventional cardiologists and stroke neurologists should borrow the heart team concept. Close collaboration between 2 different specialties in treating patients is not a novel concept in medicine. Cardiologists and cardiothoracic surgeons have long been working together since the inception of angioplasty in patients with coronary artery disease (CAD). The term “heart team” was popularized in the pivotal trial Synergy Between PCI With Taxus and Cardiac Surgery trial (SYNTAX). The SYNTAX trial paved the way for the collaboration between cardiac surgeons and interventional cardiologists in treating complex CAD. Each patient with complex CAD in the modern era is evaluated by an interventional cardiologist and a cardiac surgeon for possible percutaneous stent placement vs. coronary artery bypass graft surgery. As a result, decades of harmonious partnership between interventional cardiologists and cardiac surgeons have ensued. This unique teamwork has treated thousands of patients with complex CAD safely and effectively since the birth of angioplasty.

The efficiency and the success of the heart team approach once again was proven in transcatheter valvular therapies specifically transcatheter aortic valve replacement (TAVR). Historically, since Charles Hufnagel implanted the first artificial aortic valve and Charles Bailey and Dwight Harken performed their open commissurotomy in patients with mitral stenosis, the treatment of valvular heart disease has been exclusively been treated by cardiac surgeons. Now, patients with valvular heart disease are mandated to be seen by cardiothoracic surgeons and by an interventional cardiologist, mostly in an outpatient setting. The valve clinic was designed to deliver care to patients as fast and efficient as possible. A single visit of the patient in the valve clinic is comprised of an independent evaluation of the interventional cardiologist and a cardiac surgeon. After careful deliberation, patients are treated via either transcatheter or surgical therapies. Without question, the heart team approach is a proven concept and is now being used in the current era of transcatheter therapies.

The approach to PFO closure for stroke prevention should not be any different from the heart team concept. However, the clinical complexity of patients with PFO are completely different compared to patients with aortic stenosis (AS) and CAD. While the decision to treat patients with AS or CAD is often not a conundrum, patient selection is key in PFO closure patients. Often, it is very difficult to select patients for PFO closure since cryptogenic stroke is a diagnosis of exclusion. Only when no other etiology of ischemic stroke is evident, then closure may be indicated.

Placing a PFO closure device in a patient who does not meet the indication for closure may have drastic consequences. Prototypical PFO patients are young and healthy with many years or decades ahead of them. An implanted PFO closure device that is not indicated would expose the patient to lifelong risks of an intracardiac foreign body. To mitigate this dilemma, the decision to proceed with closure should not be decided by a single entity. The Society for Cardiovascular Angiography and Interventions (SCAI) expert consensus statement on institutional and operator requirements suggested a multi-disciplinary team composed of a stroke neurologist and an interventional cardiologist (4). The knowledge and expertise of a stroke neurologist in the diagnosis and management of PFO and stroke, especially in young, relatively healthy patients, is essential. Both entities should carefully evaluate patients not only for the indication for the procedure but also for the suitability of the patient even if it is indicated. One important goal of the heart-brain team approach is the avoidance of unnecessary and inappropriate PFO closures. The check and balance system between the two different specialties ensure that only patients with PFO-mediated strokes receive a PFO closure device after careful deliberation.

A strong PFO program must have a very rigorous selection process in order to offer the procedure to those who will benefit the most. Some institutions have already implemented a heart-brain team approach in PFO patients. The proven concept of a valve clinic for TAVR patients may be implemented in a “PFO clinic.” Stroke neurologists, general cardiologists, interventional cardiologists, interventional neurologists, electrophysiologists, hematologists, nurse practitioners, social workers, are some integral members of a heart-brain team. Bringing together expertise in all the fields in the same clinical setting allows complex clinical issues in PFO closure to be seamlessly addressed for the patients and their families in the most efficient and in the shortest amount of time.

SCAI has already established a PFO task force which includes representation from the AAN. This partnership is essential in ensuring the operator and institutional guidelines are thorough, evidence-based, and fair to both societies. Together, both societies with their members, can deliver safe and effective treatment to patients in the spirit of patient-centered care. Previously labeled as adversaries, stroke neurologists and interventional cardiologists are now considered invaluable partners in treating PFO-mediated strokes.

## Author Contributions

All authors listed have made a substantial, direct and intellectual contribution to the work, and approved it for publication.

## Conflict of Interest

The authors declare that the research was conducted in the absence of any commercial or financial relationships that could be construed as a potential conflict of interest.
